# Cognitive Neural Mechanism of Social Anxiety Disorder: A Meta-Analysis Based on fMRI Studies

**DOI:** 10.3390/ijerph18115556

**Published:** 2021-05-22

**Authors:** Xianglian Yu, Yijun Ruan, Yawen Zhang, Jiayi Wang, Yuting Liu, Jibiao Zhang, Lin Zhang

**Affiliations:** 1 Department of Education, Jianghan University, Wuhan 430056, China; psyyu@jhun.edu.cn (X.Y.); 15872393952@stu.jhun.edu.cn (J.W.); 182203301131lyt@stu.jhun.edu.cn (Y.L.); 2 Key Laboratory of Adolescent Cyberpsychology and Behavior, Ministry of Education, Key Laboratory of Human Development and Mental Health of Hubei Province, School of Psychology, Central China Normal University, Wuhan 430056, China; 3 Department of Psychology, The Chinese University of Hong Kong, Hong Kong 999077, China; ruanyijun@link.cuhk.edu.hk; 4 Department of Medical Psychology, School of Health Humanities, Peking University, Beijing 100191, China; yawen@bjmu.edu.cn

**Keywords:** social anxiety disorder, fMRI study, meta-analysis, activity likelihood estimation

## Abstract

Objective: The present meta-analysis aimed to explore the cognitive and neural mechanism of social anxiety disorder (SAD) from a whole-brain view, and compare the differences in brain activations under different task paradigms. Methods: We searched Web of Science Core Collection and other databases with the keywords related to social anxiety, social phobia, and functional magnetic resonance imaging (fMRI) for comparing persons with SAD to healthy controls and used the activation likelihood estimation method. Thirty-seven papers met the inclusion criteria, including 15 with emotional faces as stimuli, 8 presenting specific situations as stimuli, and 14 using other types of tasks as stimuli. Among these papers, 654 participants were in the SAD group and 594 participants were in the control group with 335 activation increase points and 115 activation decrease points. Results: Whole-brain analysis showed that compared with healthy controls, persons with SAD showed significantly lower activation of the left anterior cingulate gyrus (MNI coordinate: x = −6, y = 22, z = 38; *p* 0.001). Sub-group analysis based on task indicated that when performing tasks with emotional faces as stimuli, persons with SAD showed significantly lower activation of the left cerebellar slope and fusiform gyrus (MNI coordinate: x = −26, y = −68, z = −12; *p* 0.001), and significantly higher activation of the right supramarginal gyrus and angular gyrus, than healthy controls (MNI coordinate: x = 58, y = −52, z = 30; *p* 0.001). Conclusion: Individuals with social anxiety disorder show abnormal activation in the cingulate gyrus, which is responsible for the process of attention control, and task type can influence the activation pattern.

## 1. Introduction

Social anxiety disorder (SAD; previously called social phobia) has been defined as the experience of constant fear, nervousness, and avoidance in the presence of a stranger, or in social situations that involve being observed [1]. Previous studies found that social anxiety often begins in adolescence and, if left untreated, can lead to comorbidity with depression, substance abuse, and other anxiety disorders [2]. More and more researchers are using neuroimaging technology (especially magnetic resonance imaging technology) to explore the cognitive neural mechanism of SAD. This basic research may one day have applied value in the diagnosis and treatment of the disorder. 

However, not all these studies have obtained concordant conclusions. First, there is no consensus on which brain regions are related to SAD. Some researchers believed that a large-scale system of neural activity should be concerned in the diagnosis of SAD, while others considered that some distinct brain regions (e.g., right amygdala and superior temporal sulcus) are related to SAD [3,4]. Besides, researchers have no agreement on how the activities of brain regions change in SAD. For example, Gentili et al. found that the activity of the left fusiform gyrus of individuals with social phobia was significantly increased in their research, however, Frick et al. derived the opposite conclusion in their study, in which the activity of the bilateral fusiform gyrus of individuals with SAD significantly increased [5,6]. A possible explanation for inconsistent conclusions could be that there are different experimental paradigms in these studies. In previous task-state fMRI studies of SAD, researchers mostly used emotional face stimuli, social context stimuli, memory tasks, emotional Stroop tasks, and speech tasks to explore the relationships between activation of different brain structures and SAD. The results of these studies appear to differ based on task. For example, in one study using a speech task, activation of the pons, ventral striatum, amygdala, insula, and temporal polar regions of persons with SAD increased significantly, while the activations of dorsal anterior cingulate cortex and prefrontal cortex decreased significantly [7]. 

There have been four meta-analyses that included results regarding the relation between brain activation and SAD. Etkin et al. published the first meta-analysis of neuroimaging results in samples of participants with anxiety disorders, including post-traumatic stress disorder, SAD, specific phobia, obsessive-compulsive disorder, generalized anxiety disorder, and comorbid pain disorder. They concluded that there may be a general “fear circuit” centered in the amygdala and the insula, and abnormal activities in these brain regions may cause SAD [8]. Later, in 1999, Hattingh et al. published a meta-analysis exploring the affective cognition ability of persons with SAD and found that the average activations of the amygdala, temporal lobe, parahippocampal gyrus, anterior cingulate gyrus, globus pallidus, and posterior central gyrus in SAD groups were significantly lower than those in control groups [9]. Gentili et al. studied the face perception ability of patients with SAD and found that the face stimulation task led to increased activations of the amygdala, globus pallidus, superior temporal sulcus, visual cortex, and prefrontal cortex of patients with SAD [5]. Binelli et al. found abnormal activation of the limbic system in patients with SAD [10]. 

Although these meta-analyses found associations between SAD and abnormalities in certain brain regions, as a group they had limitations. Each included only seven or eight articles. They also used inconsistent methods of data analysis (some used analysis of region of interest (ROI)), the literature searches were not comprehensive, and a focus on the experimental tasks that assess the processing of emotional faces but not memory tasks, speech tasks, and situation presentations. These inconsistent results and limitations of previous studies and meta-analysis indicate that more comprehensive meta-analyses using innovative methods need to be taken to further clarify the cognitive neural mechanism of SAD. Therefore, in the current meta-analysis, we conducted a comprehensive search of the literature and used Activation Likelihood Estimation (ALE) rather than ROI to explore whole-brain activation in samples of persons with SAD when performing a range of tasks. By comparing differences in brain activation under different task paradigms within one meta-analysis, we can gain further understanding of the neural mechanism of SAD. 

Overall, the purpose of the present study was to explore the cognitive and neural mechanism of SAD from a whole-brain view and further compare differences in brain activation under different task paradigms.

## 2. Materials and Methods

### 2.1. Study Selection

**Figure 1:**
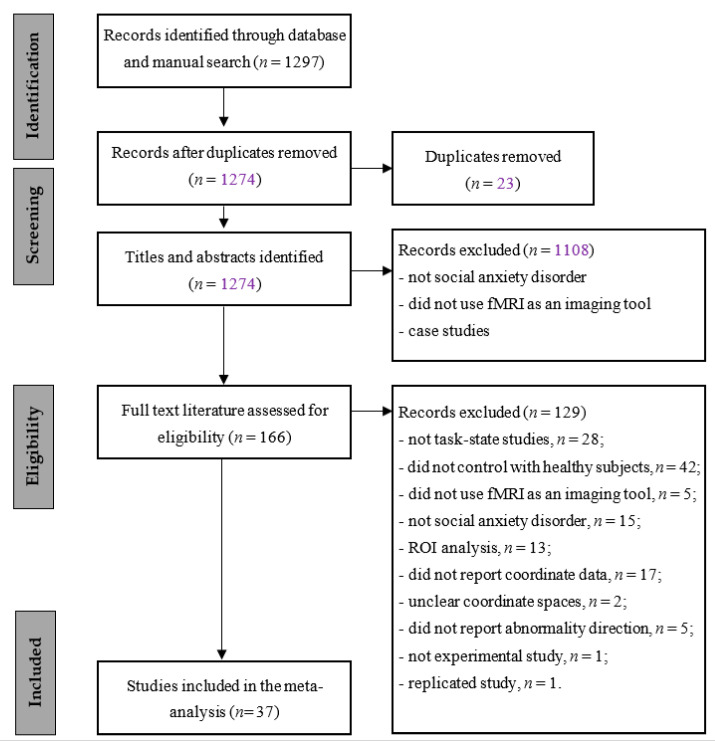
Flow diagram of literature search and selection process.

We searched databases such as Web of Science Core Collection, PubMed, and CNKI with the combination of keywords (“social anxiety” or “social phobia”) and (“functional magnetic resonance imaging [fMRI]” or “functional magnetic resonance imaging”). For the PubMed, MeSH terms were utilized. In addition, we conducted a manual search of the reference lists of papers on related topics. Reviews, meta-analyses, and case studies were excluded, although studies cited in these papers were considered for inclusion. The criteria for inclusion were as follows: (a) persons with SAD or social phobia were compared to healthy controls; (b) the study reported the results of task-state brain imaging (with no limitation on task type); (c) the imaging data were analyzed using whole-brain data analysis; (d) the study reported between-group differences in brain activation and the coordinates of that activation; and (e) the paper was written in English or in Chinese. Accordingly, the exclusion criteria were as follows: (a) persons with neither SAD nor social phobia; (b) the study did not control with healthy subjects; (c) the study did not use fMRI as an imaging tool; (d) the study did not report the results of task-state brain imaging; (e) the study utilized ROI analysis; (f) the study did not report coordinate data; (g) the study did not demonstrate clear coordinate spaces; (h) the study did not report abnormal direction; and (i) the study was not an experimental study. The procedure was conducted strictly in accordance with PRISMA guidance. See Figure 1 for the search process.

### 2.2. Data Extraction

The following data were extracted from each paper: (a) study ID, (b) author, (c) publication year, (d) age, (e) coordinates, (f) number of extracted coordinates (SAD groups healthy controls), (g) number of extracted coordinates (SAD groups healthy controls), (h) brain region, (i) gender, (j) task type, and (k) type of coordinates. Data extraction and coding were carried out by two authors independently. In order to ensure the reliability, two authors met regularly and resolved disagreements in coding and data extraction through discussion and consensus.

### 2.3. Study Quality

The quality and risk of bias (RoB) of included studies were evaluated with a modified version of the Newcastle–Ottawa scale (mNOS), which adapted to fMRI data [11]. This version uses a different set of items adapted to fMRI studies [12]. Scores on the mNOS range from 0 to 11, with 0 to 3 considered indicative of high risk, 4 to 7 as intermediate, and 8 to 11 as low risk. RoB was independently assessed by two authors. Inter-rater agreement was measured with the Kappa statistic, and disagreements were subsequently resolved by discussion with a third author.

### 2.4. ALE Meta-Analysis

Activation Likelihood Estimation(ALE) was utilized in the present study, which is a commonly used statistical method for meta-analysis in the field of neuroimaging. It uses the activation probability as an index and hypotheses concern this probability. The voxels of brain structures activated under certain conditions in each experiment included in the meta-analysis are analyzed together, and the probability of consistent activation reaching the set threshold can be calculated. In this study, meta-analyses were conducted four times in two main steps. Specifically, we conducted the overall analysis of all the data first, then the subgroup analysis (emotional face group and situational stimulus group) based on the classification of the experimental stimulus materials. The emotional faces group was the experimental paradigm with emotional faces as the stimulus material, and the situational stimulus group was the experimental paradigm with scene pictures as the stimulus material (see Table 1 for details). In this study, the GingerALE 3.0.2 software (http://www.brainmap.org/, accessed on 16 May 2019) was used for the analysis. The process is based on the parameter setting recommendations in the GingerALE instruction manual: the statistically different coordinates in the literature were extracted and transformed into spatial coordinates; the family-wise error (FWE) algorithm was employed for statistical analysis (*p* 0.001); finally, the corresponding statistical results and Figure were presented based on the Mango software program v4.1.

## 3. Results

### 3.1. Study Selection

The search produced 1297 entries (23 after removal of duplicates), 1108 of which were excluded based on the abstract, because they were (a) not social anxiety disorder, or (b) did not use fMRI as an imaging tool; or (c) case studies. The remaining 166 articles were retrieved, and full texts of them were assessed. A total of 129 articles were excluded due to (a) not being task-state studies (*n* = 28); (b) did not control with healthy subjects (*n* = 42); (c) did not use fMRI as an imaging tool (*n* = 5); (d) were not a social anxiety disorder (*n* = 15); (e) had ROI analysis (*n* = 13); (f) did not report coordinate data (*n* = 17); (g) had unclear coordinate spaces (*n* = 2); (h) did not report abnormality direction (*n* = 5); (i) were not an experimental study (*n* = 1); (j) were a replicated study (*n* = 1). A total of 37 articles were included in the meta-analysis, as described in the PRISMA flow diagram (see Figure 1). 

### 3.2. Characteristics of the Included Studies

A total of 1297 publications were retrieved from initial search, among which 37 studies met the inclusion criteria (654 participants in the SAD groups and 594 participants in the control groups). There were 15 studies that adopted emotional faces as task stimuli [6,10,13,14,15,16,17,18,19,20,21,22,23,24,25], 8 that presented specific situations as task stimuli [26,27,28,29,30,31,32,33], and the other 14 employed other types of tasks [7,34,35,36,37,38,39,40,41,42,43,44,45,46]. In total, the coordinates where SAD groups performed significantly better or significantly worse than control groups were 335 and 115 respectively (see specific characteristics in Table 1).

### 3.3. Study Quality

The result showed that the overall Cohen kappa (mean ± SD) was 0.953 ± 0.08 ranging from 1 to 0.73. Consensus and Cohen kappa for each item of the mNOS are reported in Table S2. The lower agreement was for drop-out rate (0.78) and false positive correction (0.73). Twenty-six studies were considered as low RoB, eleven as intermediate risk, and none as high risk of bias. A detailed description of the quality of each study is presented in the Supplementary Materials. 

### 3.4. Activation Likelihood Estimation

**Figure 2:**
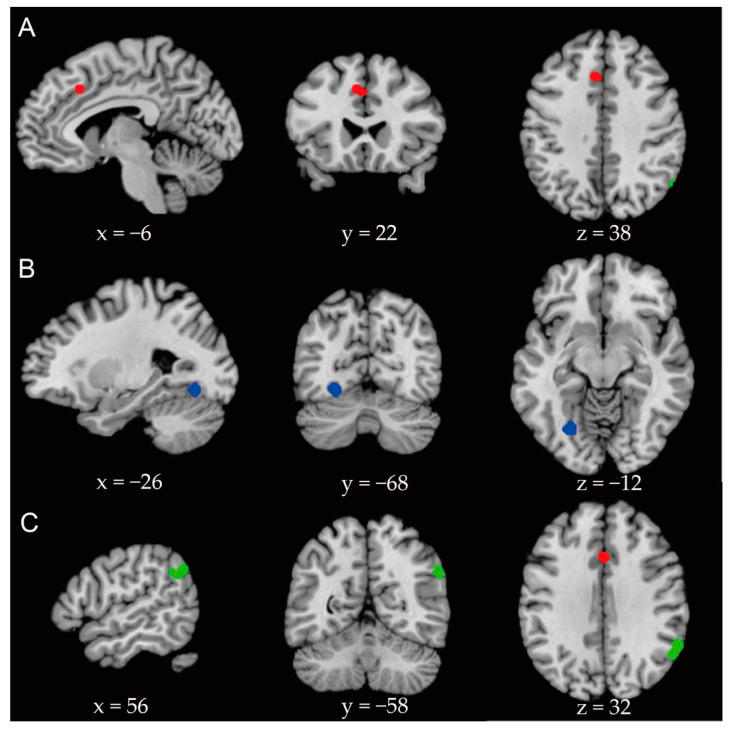
Brain regions with significantly lower activation in SAD groups than control groups (*p* 0.001). Red areas (**A**) illustrate the results of the whole-brain analysis, blue areas (**B**) illustrate the brain regions with significantly lower activation in the SAD groups than control groups during emotional face stimuli tasks, and green areas (**C**) illustrate the brain regions with significantly lower activation in the SAD groups than control groups during situational stimuli tasks.

The whole-brain analysis showed that the activation of the left cingulate gyrus (MNI coordinates: x = −6, y = 22, z = 38) in SAD groups was significantly lower than that in control groups (maximum ALE value = 0.015). The results of sub-group analysis showed that in studies using tasks with emotional faces as stimuli, the activation of the left cerebellar slope (extending to the fusiform gyrus; MNI coordinates: x = −26, y = −68, z = 12) in the SAD groups was significantly lower than in the control groups (maximum ALE value = 0.015), and there were no clusters showing higher activation in the SAD groups. In tasks with specific situations as stimuli, the brain area with significantly lower activation in the SAD groups was the upper right marginal gyrus (extended to the angular gyrus; MNI coordinates: x = 58, y = −52, z = 30; maximum ALE value = 0.013). There were no other brain regions with significantly higher activation in the SAD groups (see Table 2 and Figure 2 for details). 

## 4. Discussion

In this meta-analysis, we used the ALE method to analyze brain functional imaging data of people with SAD and healthy controls. We found that the activation of the left anterior cingulate cortex, cerebellar slope and fusiform gyrus, right superior marginal gyrus, and angular gyrus were lower in SAD groups than in healthy control groups. In this section, we first discuss the abnormal activation of brain regions in the SAD groups, then discuss the influence of different experimental paradigms on the activation of brain regions in the SAD groups, and finally explain the advantages and limitations of this study.

### 4.1. Anterior Cingulate Cortex

The anterior cingulate gyrus belongs to the medial prefrontal cortex (mPFC) group. It is an important structure of the limbic system and plays an important role in the generation and regulation of emotions. The cingulate gyrus is the main brain area that helps information from focused attention enter the conscious level, and its activation state has been confirmed in various experiments [47]. For example, in simple and easy processing, the anterior cingulate gyrus is less able to cope with selective attention, and the posterior cingulate gyrus is less able to promote the execution of appropriate responses and/or inhibit the execution of unsuitable responses. These weakening effects may result in a decline in the anti-interference ability of individuals with SAD, making them feel more anxious. 

The anterior cingulate gyrus is also an important connection node between the prefrontal cortex and limbic system. It plays an important role in perceiving and processing social rejection, and research on the activity of this structure provides evidence that social pain and physical pain share a common neurocognitive function [48]. The anterior cingulate gyrus is also important in a person’s coping with social stressors [49]. For example, Wang et al. found that a reduction in the connection between the left anterior cuneate/posterior cingulate and the gyrus-anterior cingulate gyrus in anxious patients will lead to weakened emotion regulation, and finally cause anxiety [50].

### 4.2. Angular Gyrus/Supramarginal Gyrus

The meta-analysis found that the angular gyrus activity was significantly lower in individuals with SAD than in healthy controls when performing a situational stimulus task. The angular gyrus plays a very important role in mental constructs such as thoughts, feelings, and beliefs related to oneself and others [51]. Qiu et al. reported that during the resting state without external stimuli, individuals with SAD had emotional and attentional deviations and distorted negative self-belief [52]. The results of this study support previous researchers’ conclusions that individuals who hold negative beliefs about themselves show abnormal angular gyrus activity [53]. We also found that the activity of the upper right supramarginal gyrus of individuals with SAD was significantly lower than that of healthy controls in situational stimulus tasks. Previous studies reported that when individuals with SAD recognized their own faces, they also showed significant reductions of activity in the supramarginal gyrus. Therefore, some researchers believe that the cognitive bias of individuals with SAD when performing a situational stimulus task may be related to the cognitive distortion of their own faces [54].

### 4.3. Cerebellar Slope/Fusiform Gyrus

This meta-analysis showed that the activation of the left cerebellar slope of individuals with SAD was significantly lower than that of the healthy controls when performing face recognition tasks. The cerebellum is an important part of the motor network, and structurally it is closely connected with the limbic system. It is also an important part of emotional processing [55]. Previous studies found that the cerebellar area showed abnormal changes when individuals with SAD spoke in public, watched angry faces, performed confrontational computing tasks, and were exposed to different social tasks. The results of these previous studies are consistent with those of the current research [56].

The meta-analysis also showed that there was significantly lower activity of the fusiform gyrus in the SAD groups than in the healthy control groups during emotional faces tasks. This may indicate that the individuals with SAD adopted an avoidance strategy and reduced their fixation on emotional facial stimuli. The results of several previous studies support this finding. For example, Gentili et al. found that the activity of the left fusiform gyrus of individuals with social phobia was significantly reduced when watching emotional and neutral faces, compared with watching garbled pictures [5]. However, other studies reported opposite results. For example, Frick et al. found that the activity of the bilateral fusiform gyrus of individuals with SAD significantly increased when they looked at scared faces [6]. A reasonable explanation for this inconsistency is that the activity of the fusiform gyrus depends on whether individuals with SAD adopt avoidance strategies, but also on the type of research paradigm the study used. Future research could use eye tracking to test the hypothesis that individuals with SAD avoid looking at the emotional face, leading to reduced activity in the fusiform gyrus.

### 4.4. Different Task Types Affect Activation Patterns in Brain Regions

In the emotional face stimulation task, previous studies found abnormal activity in the limbic system of individuals with SAD [8]. Specifically, compared to healthy controls, people with SAD showed significantly higher activity in the amygdala, sulcus, and the parahippocampal gyrus when viewing angry and contemptuous faces. The hippocampus’ response to emotional faces is positively correlated with the severity of social anxiety symptoms [13]. This meta-analysis excluded studies that only analyzed a specific brain region of interest and studies that did not report the coordinates of the brain regions showing abnormal activity. Using these exclusion criteria, we found a significant decrease in the activation of the left cerebellar slope, providing new insights for research in this field. That is, SAD may involve dysfunction of a wide range of neural networks, including the limbic system and cerebellum.

In the specific situational stimulus paradigm, the activation of the upper right supramarginal gyrus in the SAD groups was significantly lower than that of the control groups. The upper right supramarginal gyrus plays an important role in regulating empathy for others. When this area works abnormally, people are unable to make rapid judgments about other people’s emotions and have difficulty feeling empathy. Dysfunction in this area can also lead to more self-centeredness because of high levels of cognition at the expense of emotion, known as intellectualization [57]. Previous research found that people with SAD were not incapable of recognizing other people’s emotions, but they still had low ability for empathy. One reason may be that they have problems with mentalization [58]. Compared with emotional facial stimuli, certain situational stimuli may be more likely to create a sense of an interpersonal environment, which serves as an important condition for the creation of empathy [29].

### 4.5. Advantages and Limitations of This Study

This meta-analysis study adopted the ALE method and conducted whole-brain analysis and sub-group task analysis in studies comparing individuals with SAD to healthy controls. These methods make it likely that the results are more reliable than those reported in earlier meta-analyses in this literature. However, the present study also has some shortcomings. The first limitation is that the number of studies included in the analyses was relatively small. We had intended to compare the results from studies using a range of experimental tasks but found that most of the studies used either the situational stimulation task or the emotional face stimulation task, and the other studies could not be characterized as sharing a certain paradigm. We expect further relevant sub-group studies to stabilize the current results. Second, the meta-analysis method examines many different studies, which may reduce the homogeneity of the data and affect the stability of the research results. Third, because of the lack of behavioral data of some studies, we did not explore the relationship between behavioral data and activated brain regions by using regression analysis, which may limit the generalizability of the results. Finally, in this study, no functional abnormalities in other brain areas such as the amygdala were found, which may have resulted from the use of whole brain analysis.

## 5. Conclusions

Overall, the present meta-analysis utilized a reliable method (i.e., ALE) to conduct whole-brain analysis, and found some brain activations were lower in SAD groups than in healthy control groups, which revealed the neurological mechanisms of the SAD groups. People with SAD mainly show abnormal activation in the cingulate gyrus, which is related to attention control. Besides, the present study also found that brain activations were different between experimental paradigms in the SAD groups, that is, task types can affect the activation pattern. This finding indicated that the results should be interpreted with caution due to the different experimental paradigms.

## Figures and Tables

**Table 1:** Characteristics of the included studies.

ID	Ref. ID	Study Year	Number of Participants SAD/Healthy Controls	Number of Extracted Coordinates (SAD Groups) Healthy Controls)	Extract Number of Coordinates (SAD Groups Healthy Controls)	Coordinate System	Task Type
1	[13]	2002	15/15	8	0	Talairach	Emotional face
2	[14]	2005	11/11	40	0	Talairach	Emotional face
3	[15]	2006	10/10	8	0	Talairach	Emotional face
4	[16]	2008	11/11	10	2	MNI	Emotional face
5	[17]	2009	8/7	2	0	Talairach	Emotional face
6	[18]	2010	12/12	12	1	MNI	Emotional face
7	[19]	2010	8/7	11	4	Talairach	Emotional face
8	[20]	2012	29/26	14	0	MNI	Emotional face
9	[21]	2012	18/18	19	6	MNI	Emotional face
10	[6]	2013	14/12	9	1	MNI	Emotional face
11	[22]	2013	27/27	0	11	Talairach	Emotional face
12	[23]	2014	23/24	0	1	MNI	Emotional face
13	[10]	2016	20/20	0	8	MNI	Emotional face
14	[24]	2017	12/13	9	0	MNI	Emotional face
15	[25]	2016	19/21	4	1	MNI	Emotional face
16	[26]	2009	27/27	20	27	Talairach	Specific situations
17	[27]	2009	15/17	14	26	Talairach	Specific situations
18	[28]	2009	11/11	5	0	MNI	Specific situations
19	[29]	2011	6/9	0	5	MNI	Specific situations
20	[30]	2013	20/20	2	6	MNI	Specific situations
21	[31]	2014	20/20	15	0	Talairach	Specific situations
22	[32]	2016	30/30	12	0	Talairach	Specific situations
23	[33]	2017	24/24	11	0	Talairach	Specific situations
24	[7]	2004	8/6	3	6	Talairach	Speech task
25	[34]	2014	17/17	2	1	Talairach	Speech task
26	[35]	2018	51/13	2	1	MNI	Speech task
27	[36]	2008	12/12	1	0	Talairach	Listen to the words and recognize the emotional colors
28	[37]	2010	16/16	5	0	Talairach	Story reading
29	[38]	2011	16/18	21	2	Talairach	Emotional pictures
30	[39]	2011	15/15	10	0	Talairach	Discourse presentation
31	[40]	2012	15/15	2	0	MNI	Cognitive task
32	[41]	2011	20/20	2	0	Talairach	Security review awareness task
33	[42]	2014	21/23	8	0	MNI	Emotion regulation task
34	[43]	2015	16/16	6	0	Talairach	Emotion Stroop task
35	[44]	2016	20/20	15	0	MNI	Memory task
36	[45]	2017	21/22	33	3	MNI	Currency delayed
37	[46]	2017	16/16	0	3	Talairach	Time estimation task
	Total		654/594	335	115		

**Table 2:** The coordinates of brain regions with significantly lower activation in SAD groups compared to healthy control groups.

	Central Coordinates			Volume mm^3^	*p*–Value	Maximum ALE Value	Cerebral Area
	X	Y	Z				
Global analysis	−6	22	38	584	0.000124	0.015	Anterior cingulate gyrus
Emotional face task	−26	−68	−12	496	0.00000034	0.015	Cerebellar slope, fusiform gyrus
Situational task	58	−52	30	784	0.0000046	0.013	Supramarginal gyrus and angular gyrus

## Data Availability

Not applicable.

## Supplementary Materials

The following are available online at https://www.mdpi.com/article/10.3390/ijerph18115556/s1, Table S1: data coding for all included studies; Table S2: Study quality evaluated by using modified Newcastle-Ottawa Scale (mNOS).
