# Supplementary material for: Cognitive Neural Mechanism of Social Anxiety Disorder: A Meta-Analysis Based on fMRI Studies
Source: Int J Environ Res Public Health. 2021 May 22;18(11):5556. doi: 10.3390/ijerph18115556 (PMC8196988; doi:10.3390/ijerph18115556)
Supplement: Supplementary file 1 [file ijerph-18-05556-s001.zip › ijerph-1193894-SI.pdf]

Table S1 data coding for all included studies

ID	Authors	Year	Brain regions	Central coordinates	Number of extracted coordinates (SAD groups > healthy controls)	Extract number of coordinates (SAD groups < healthy controls)	Number of participants (SAD/healthy controls)	Gender (M/F)	Age（SAD/HC）	Stimulus Task
1	Blair, K. S., Geraci, M., Hollon, N., Otero, M., DeVido, J., Majestic, C., . . . Pine, D. S	2010	Increase: Left ventromedial prefrontal gyrus; Left dorsomedial prefrontal gyrus; Left insula; Right insula; Right amygdala/parahippocampal gyrus	Increase: –13 60 16; –23 32 34; –37 –14 0; 26 16 –6; 41 –8 –18	5	0	16/16	9:7/7:9	35.1 (9.60)/30.0(8.37)	Story reading
2	Stein, M. B., Goldin, P. R., Sareen, J., Zorrilla, L. T., & Brown, G. G	2002	Increase: MFC; IFG; SFG; LA; LU; LU; RU; LPHG	Increase: 2 55 35; 44 31 3; 18 60 27; 20 5 22; 15 8 22; 18 5 25; 22 3 29; 20 7 21	8	0	15/15	10:5/10:5	None	Emotional face

3	Amir, N., Klumpp, H., Elias, J., Bedwell, J. S., Yanasak, N., & Miller, L. S	2005	Increase: Anterior cingulate gyrus; Caudate; Insula; Lingual gyrus; Middle frontal gyrus; Parahippocampal gyrus; Posterior cingulate gyrus; Posterior cingulate; Superior temporal gyrus; Supramarginal gyrus; Vermis of cerebellum; Frontal lobe, subgyral; Inferior frontal gyrus; Middle frontal gyrus; Middle temporal gyrus; Superior occipital gyrus; Superior temporal gyrus; Angular gyrus; Anterior cingulate gyrus; Anterior cingulate; Inferior frontal gyrus; Middle frontal gyrus; Middle occipital gyrus; Middletem poral gyrus; Posterior cingulate gyrus; Precuneous; Superior occipitalgyrus; Thalamus	Increase: 6 17 25; -8 42 16; 12 39 13; -12 -9 23; -4 22 6; -30 -13 14; 2 -60 12; 38 37 9; -26 -39 -5; 10 -32 26; 8 -38 15; -42 -12 -4; 44 -30 27; 2 -58 -2; 24 27 6; -20 32 15; -44 39 0; -36 35 4; -42 40 15; 44 -61 29; -38 -61 21; 28 -73 20; -44 -36 9; -34 -53 27; -12 33 -2; 6 30 11; 24 26 10; -50 9 24; 34 19 -13; 38 21 -6; 18 43 -4; -34 -81 11; 40 -60 7; 14 -37 31; -18 -53 28; -18 -65 25; -22 -21 10; -14 -8 2; -22 -21 10; 12 -10 0;	40	0	11/11	73%Female	24.1(5.2)/23.9(5.7)	Emotional face
4	Phan, K. L., Fitzgerald, D. A., Nathan, P. J., & Tancer, M. E	2006	Increase: right amygdala; right amyg dala; amygdala; right postcentral sulcus; left parahippocampal gyrus; right dorsal anterior cingulate cortex;	Increase: 26, -9, -20; 26, - 8, -20; 18, -5, -16; 30, - 5, -24; 20, -5, -17; 50, -12, 18; -26, - 21, -20; 6, 3, 36;	8	0	10/10	5:5/5:5	26.7 (6.8)/26.6(6.8)	Emotional face
5	Evans, K. C., Wright, C. I., Wedig, M. M., Gold, A. L., Pollack, M. H	2008	Increase: Dorsal amygdala; Supramarginal gyrus; Superior frontal cortex; Lingual gyrus; Fusiform gyrus; Precentral gyrus; Cerebellum crus II; Insular cortex; Posterior cingulate gyrus; Middle temporal gyrusDecrease: Anterior cingulate gyrus; Middle frontal gyrus	Increase: 26 -6 -8; -60 -55 28; -10 0 66; -24 -82 -2; -24 -68 6; -40 -64 0; 34 -64 -6; 34 -12 48; 14 -76 -36; 44 -12 14; -16 -26 44; 58 -22 -14; Decrease: 18 26 24; 44 42 30;	10	2	11/11	4:7/4:7	29.0(7.5)/27.9(10.6)	Emotional face
6	Gentili, C., Ricciardi, E., Gobbini, M. I., Santarelli, M. F., Haxby, J. V., Pietrini, P., & Guazzelli, M	2009	Increase: Posteriorcingulate/precuneus; Posterior cingulate/precuneus	Increase: 19 −74 38; −3 −78 48	2	0	8/7	4:4/4:3	39(7)/30(7)	Emotional face

7	Klumpp, H., Angstadt, M., Nathan, P. J., & Phan, K. L	2010	Increase: Parahippocampal gyrus; Inferior frontal gyrus; Orbital frontal cortex; Insula; Cerebellum; Midbrain; Putamen; Cuneus; Thalamus; Parahippocampal gyrus; Insula; Decrease: Middle frontal gyrus	Increase: -15 9 -9; 24 6 -15; -3 18 -21; -42 21 9; 33 78 -42; 48 -60 -45; 6 -24 -12; -18 9 -6; 15 -78 21; -18 -18 6; 12 -9 -21; -36 -15 6; Decrease: 39 42 9	12	1	12/12	None	28.2(8.6)/33.6(9.6)	Emotional face
8	Danti, S., Ricciardi, E., Gentili, C., Gobbini, M. I., Pietrini, P., & Guazzelli, M	2010	Increase: Cing; PreCun; SM; SM; IPL; IPS; IFG; IFG; PreCun; IFG; SM; Decrease: Ant MPFC; S2; STS	Increase: 7 −60 6; 14 −58 18; −59 −6 14; −60 −4 16; −34 −51 38; −26 −49 43; −63 −5 14; 55 −8 14; −25 −64 39; 35 25 −16; 5 −42 53; Decrease: 41 42 22; 46 −40 22; 43 −25 30; 54 −48 10	11	4	8/7	4:4/4:3	39 (7)/30(7)	Emotional face
9	Klumpp, H., Angstadt, M., & Phan, K. L	2012	Increase: left aINS; right aINS; Inferior frontal gyrus; Insula; Parietal superior gyrus; Caudate; Postcentral gyrus; Dorsal medial frontal cortex; Parahippocampal gyrus; Temporal inferior gyrus; Amygdala	Increase: −40, 20, −4; 38, 24, −4; 58 10 36; −54 16 32; −40 20 −4; 38 24 −4; −28 −68 52; −4 0 10; −52 −22 36; −2 24 46; 24 −54 −8; 48 −48 −30; −22 −12 −12; 14 2 −20	14	0	29/26	17:12/16:10	24.7(5.9)/26.2(6.3)	Emotional face

10	Labuschagne, I., Phan, K. L., Wood, A., Angstadt, M., Chua, P., Heinrichs, M., . . . Nathan, P. J	2012	Increase: Calcarinef issure; Precuneus; Supplementary motor cortex; Anterior cingulate cortex; Medial prefrontal cortex; Lingual gyrus/cerebellum; Cuneus; Superior temporal cortex; Postcentral gyrus; Middle occipital/middle temporal gyrus; Mid-cingulate cortex; Supplementary motor cortex; Precentral gyrus; Middle occipital gyrus/cuneus; Angular gyrus/ middle temporal gyrus; Cerebellum; Inferior temporal cortex; Decrease: Medial prefrontal/anterior; cingulate cortex; Operculum; Inferior temporal gyrus	Increase: -16 -62 16; 16 -56 14; 10 -16 62; -16 58 10; -12 56 -4; 2 66 6; 14 56 -4; -12 -46 -10; -6 -98 16; 54 -30 10; 26 -40 74; 44 -74 24; -6 8 24; 16 -20 54; -24 -30 54; -24 -100 -10; 54 -72 30; 50 -58 -30; -60 -58 -18; Decrease: 2 26 -4; 10 48 -14; 14 60 36; -10 52 30; -44 -6 16; -44 -6 -34;	19	6	18/18	Male	29.4(9.0)/29.9(10.2)	Emotional face
11	Frick, A., Howner, K., Fischer, H., Kristiansson, M., & Furmark, T	2013	Increase: Fusiform gyrus, right; Fusiform gyrus, left; Precentral gyrus,left; Superior frontal gyrus,left; Cerebellum,right; Cerebellum,left; Amygdala, right; Decrease: vmPFC, left	Increase: 45 -52 -20; -42 -67 -20; -27 14 37; -18 47 37; 60 -49 4; 42 -64 -23; -27 -73 -44; -39 -70 -29; 24 -10 -11; Decrease: -15 35 -8;	9	1	14/12	None	32.4(8.8)/28.0(8.2)	Emotional face
12	Ziv, M., Goldin, P. R., Jazaieri, H., Hahn, K. S., & Gross, J. J	2013	Decrease: Left inferior frontal gyrus; Dorsal anterior cingulate gyrus; Left lateral orbitofrontal cortex; Left fusiform gyrus; Right cerebellum; Right fusiform gyrus; Left putamen; Left lingual gyrus; Right cerebellum; Right cerebellum; Left superior temporal gyrus BA41	Decrease: -34, 25, 15; 0, 18, 33; -31, 52, -6; -24, -68, -12; 3, -68, -9; 24, -75, -12; -28, -13, -6; -7, -75, -12; 24, -51, -16; 17, -58, -12; -58, -13, 12	0	11	27/27	15:12/14:13	31.1(7.6)/32.6(9.5)	Emotional face

13	Wheaton, M. G., Fitzgerald, D. A., Phan, K. L., & Klumpp, H	2014	Decrease: midbrain;	Decrease: (6,−32,−6);	0	1	23/24	69.6%F/54.2%F	26.1(6.7)/25(5.6)	Emotional face
14	Binelli, C., Muniz, A., Subira, S., Navines, R., Blanco-Hinojo, L., Perez-Garcia, D., . . . Martin-Santos, R.	2016	Decrease: cluster（the left orbitofrontal cortex, left amygdala, left stratium and left caudate）; Left medial prefrontal cortex; Left lateral prefrontal cortex; Right fusiform gyrus; Left fusiform gyrus; Left precuneus; Right lateral temporal cortex; Left lateral temporalcortex; Cerebellum	Decrease: −8, 18, 56; −42, 24, 24; 30, −74, −16; −28, −74, −10; −4, −68, 42; 68, −28, −10; −62, −44, −14; 12, −56, −46	0	8	20/20	14:6/12:8	25.05(5.25)/25.65(6.31)	Emotional face
15	Michalowski, J. M., Matuszewski, J., Drozdziel, D., Koziejowski, W., Rynkiewicz, A., Jednorog, K., & Marchewka, A	2017	Increase: Superior Frontal Gyrus; Middle Cingulate Gyrus; Precuneus; Superior Temporal Gyrus; Middle Temporal Gyrus; Fusiform Gyrus; Anterior Orbital Gyrus	Increase: 18 −2 68; −14 −5 68; −24 14 65; −2 2 27; −5 −66 60; 47 −5 −23; 62 −53 −2; 35 −75 −17; −29 36 −12	9	0	12/13	6:6/6:7	Mean of all participants 22.8	Emotional face
16	Tadayonnejad, R., Klumpp, H., Ajilore, O., Leow, A., & Phan, K. L	2016	Increase: Right lateral orbitofrontal cortex; Left superior frontal gyrus; right middle occipital gyrus; Left superior occipital gyrus Decrease: Left middle occipital gyrus	Increase: 36 62 -6; -4 -20 82; 32 -98 6; -20 -77 42Decrease -28 -98 10	4	1	19/21	10:9/8:13	26.95(8.11)/26.91(5.5)	Emotional face

17	Goldin, P. R., Manber-Ball, T., Werner, K., Heimberg, R., & Gross, J. J	2009	Decrease: L dorsolateral PFC; R dorsolateral PFC; R dorsolateral PFC; L superior temporal gyrus; L superior temporal gyrus; L posterior cingulate cortex; R supramarginal gyrus; L supplemental motor area; R inferior frontal gyrus; Dorsomedial PFC; L ventrolateral PFC; Dorsal anterior cingulate cortex; L inferior frontal gyrus; L dorsolateral PFC; R dorsolateral PFC; Medial anterior PFC; Medial anterior PFC; R dorsolateral PFC; L superior temporal gyrus; L posterior middle temporal gyrus; R inferior temporal gyrus; R supramarginal gyrus, R inferior parietal lobule; Medial precuneus; L superior parietal lobule, L inferior parietal lobule; R inferior parietal lobule; L thalamus; L thalamus; Increase: L inferior parietal lobule; R inferior parietal lobule; R dorsolateral PFC; R ventrolateral PFC; L dorsolateral PFC; R dorsolateral PFC; R dorsolateral PFC; L insula; L anterior insula; R insula; R posterior insula; L precentral gyrus; L inferior parietal lobule; L inferior parietal lobule; R precuneus; L precuneus; R superior temporal gyrus; R thalamus; L thalamus; R lentiform nucleus, putamen	Decrease: -41 7 36; 41 1 36; 52 18 36; -69 -13 8; -65 -6 1; -7 -20 29; 58 -51 32; -14 -10 50; 55 21 -2; 0 21 63; -34 62 -9; 3 14 29; -34 28 -2; -48 7 36; 48 18 36; -3 62 36; 14 62 19; 52 25 22; -69 -13 8; -31 -75 22; 55 -34 -16; 55 -58 32; -3 -68 43; -38 -65 53; 48 -58 46; -7 -10 -2; -14 -20 -2; Increase: -58 -34 36; 48 -51 50; 48 11 39; 28 69 12; -41 38 26; 17 28 56; 41 38 32; -41 7 8; -31 18 8; 38 4 -2; 34 -17 19; -65 1 22; -58 -31 32; - 58 -48 39; 10 -58 36; -10 -37 50; 55 -6 5; 7 -10 12; -10 -13 12; 17 4 -5	20	27	27/27	15:12/15:12	32.1(9.2)/32.2(9.5)	Specific situations

18	Goldin, P. R., Manber, T., Hakimi, S., Canli, T., & Gross, J. J	2009	Increase: Medial OFC; L subgenual ACC; L parahippocampal gyrus; R parahippocampal gyrus; L postcentral gyrus; L postcentral gyrus; L superior parietal lobule; L middle occipital gyrus; R inferior occipital gyrus; R lingual gyrus; R cuneus; R mid-dorsolateral PFC; R lentiform/caudate; L lentiform/caudate; Decrease: L medial precuneus; L inferior parietal lobule; R supramarginal gyrus; Medial PFC; Supragenual ACC; L middle frontal gyrus; R posterior insula; R precentral gyrus; Medial cuneus; R lingual gyrus; L lingual gyrus; R postcentral gyrus; R posterior cingulate, cuneus; R superior parietal lobule; R superior parietal lobule; L inferior parietal lobule; R posterior cingulate; L superior parietal lobule; R fusiform gyrus; L superior temporal gyrus; R superior temporal gyrus; L fusiform gyrus; R superior temporal gyrus; R middle frontal gyrus/ premotor cortex; L superior temporal gyrus; L superior temporal gyrus	Increase: −8 49 −9; −13 25 −12; −21 −27 −6; 19 −28 −3; −55 −24 56; −43 −17 49; −21 −55 67; −40 −74 18; 24 −89 −5; 10 −55 −2; 20 −79 28; 21 49 29; 10 4 −6; −10 −4 −6; Decrease: 7 −54 35; −49 −55 48; 60 −54 29; 7 51 8; 10 −6 43; −24 −10 46; 41 −20 5; 41 −20 60; −7 −89 32; 10 −89 5; −21 −61 1; 45 −20 53; 14 −58 8; 21 −65 63; 31 −48 63; −41 −37 50; 17 −58 15; −21 −48 70; 41 −55 −19; −58 −17 12; 62 −17 15; −24 −68 −19; 65 −13 5; 58 4 36; −62 −20 5; −52 −27 12	14	26	15/17	6:9/8:9	31.6 (9.7)/32.1 (9.3)	Specific situations
19	Shah, S. G., Klumpp, H., Angstadt, M., Nathan, P. J., & Phan, K. L	2009	Increase: right amygdala; bilateral insula	Increase: 36,4,–28; −26,–10,–14; 36,–6,4; [48,8,6]; −26,10,6	5	0	11/11	8:3/6:5	27.45(8.96)/30.55(7.69)	Specific situations
20	Nakao, T., Sanematsu, H., Yoshiura, T., Togao, O., Murayama, K., Tomita, M., . . . Kanba, S	2011	Decrease: Left PCC; Left PCC; Left cerebellum; Left precuneus; Right PCC	Decrease: −12 −48 18; −12 −26 32; −10 −64 34; −4 −58 44; 8 −44 24	0	5	6/9	4:2/6:3	31.7(7.9)/32.8(5.0)	Specific situations

21	Carles Soriano-Mas(Jesus Pujol)	2013	Increase: Primary Visual Cortex; Decrease: R Prefrontal Cortex; L Parietal Cortex; R Parietal Cortex; Medial Frontal Cortex; L Prefrontal Cortex; R Prefrontal Cortex	Increase: 10, -98,12; 4, -96,6; Decrease: 38,24,52; -54, -62,46; 62, -62,42; 6,32,34; -18,34,30; 42,28,36	2	6	20/20	5:15/6:14	24.2(5.2)/24.4 (5.6)	Specific situations
22	Boehme, S., Mohr, A., Becker, M. P., Miltner, W. H., & Straube, T	2014	Increase: left amygdala; anterior cluster; mid-insula cluster; right insula; Superior frontal gyrus (BA 10); Middle frontal gyrus (BA 46); Inferior frontal gyrus (BA 44); Superior frontal gyrus (BA 8); Superior frontal gyrus (BA 9); Globus pallidus; Inferior parietal gyrus (BA 40); Inferior temporal gyrus (BA 20)	Increase: -23 0 -19; -24 23 13; -36 5 16; 36 20 13; 42 -1 13; 14 21 29; 9 27 29; 23 62 27; -50 24 19; 57 11 10; -15 50 39; 19 60 24; 15 -1 6; 55 -48 42; 46 -7 -34	15	0	20/20	10:10/10:10	23.85/24.20	Specific situations
23	Heitmann, C. Y., Feldker, K., Neumeister, P., Zepp, B. M., Peterburs, J., Zwitserlood, P., & Straube, T	2016	Increase: L angular gyrus; L cerebellum lobule V; L dmPFC; L lPFC; L middle temporal gyrus; L PCC; L PCC; L precentral gyrus; L precuneus; L globus pallidus/putamen; R SPL; L thalamus	Increase: -44 -75 24; -11 -42 -13; -6 40 33; -26 55 3; -55 -39 -2; -3 -41 35; -11 -54 25; -40 4 35; -11 -55 48; -22 -1 2; 15 -53 58; -5 -12 -1	12	0	30/30	None	27.5(7.74)/27.07(5.35)	Specific situations

24	Heitmann, C. Y., Feldker, K., Neumeister, P., Brinkmann, L., Schrammen, E., Zwitserlood, P., & Straube, T	2017	Increase: L Insula (BA13); L superior temporal sulcus (BA22); R Precuneus (BA7) ; R Temporal lobe (BA13) ; R Parahippocampal gyrus; R inferior frontal gyrus (BA45); R inferior frontal gyrus (BA47); R dorsomedial prefrontal gyrus (BA32) ; R dorsolateral prefrontal cortex (BA8) ; R dorsolateral prefrontal cortex (BA6)	Increase: −37 12 15; 4 −53 48; 46 −46 19; -61 -46 15; 43 -26 -5; 36 24 3; 5 36 46; 8 20 42; 46 12 29; 39 6 46; 17 3 55	11	0	24/24	7：17/7:17	27.29(7.69)/27.38 (5.77)	Specific situations
25	Lorberbaum, J. P., Kose, S., Johnson, M. R., Arana, G. W., Sullivan, L. K., Hamner, M. B., . . . George, M. S	2004	Increase: L Amygdala/uncus/; parahippocampus/hippocampus; LTemporal pole; Decrease: L Dorsal anterior cingulate (BA 24,32)/MPFC (BA 8, 32)/DLPFC (BA 9)	Increase: -16 0 -16; -12 0 -24; -44 4 -12; Decrease: -24 24 24; - 20 4 28; -8 20 40; -24 16 36; -8 48 36; -12 36 40	3	6	8/6	None	33.8(2.8)/35.07(3.9)	Speech task
26	Boehme, S., Ritter, V., Tefikow, S., Stangier, U	2014	Increase: right insula; right amygdala; Decrease: ventral striatum;	Increase: 47 -3 5; 26 -2 -8; Decrease: -4 10 6	2	1	17/17	None	31.12(10.52)/30.82(8.63)	Speech task

27	Bunford, N., Kujawa, A., Fitzgerald, K. D., Monk, C. S., & Phan, K. L	2019	Increase: Middle Occipital Gyrus/Fusiform; Insula; Decrease: Middle Temporal Gyrus	Increase: 24 -85 10; 39 7 -17; Decrease-54 -49 7	2	1	51/13	11:24/5:8	27.3/29.1	Speech task
28	Quadflieg, S., Mohr, A., Mentzel, H. J., Miltner, W. H., & Straube, T	2008	Increase: right OFC;	Increase: 26, 51, -2;	1	0	12/12	6:6/6:6	23.25/24	Listen to the words and recognize the emotional colors
29	Bruhl, A. B., Rufer, M., Delsignore, A., Kaffenberger, T., Jancke, L., & Herwig, U	2011	Increase: MPFC/MFG R; DLPFC/SFG L; Dorsal cingulate L; STG R; MTG R; Temporal pole R; IPS L; Superior occipital gyrus R; Med. occipitotemporal gyrus R; Med. occipitotemporal/ lingual gyrus L; Inf. occipital gyrus L; Fusiform gyrus L; Dorsal thalamus/pulvinar/upper midbrain R; Med. thalamus bilat.; Parahippocampal gyrus L; Caudate body L; Amygdala L; TOC R; Fusiform gyrus R; Fusiform gyrus L; MTG L; Decrease: VLPFC/IFG/OFC L; VLPFC/SFG/IFG/OFC L	Increase: 6 35 31; −12 20 46; −18 −34 34; −51 −31 7; 51 −58 1; 48 11 −11; −51 −52 37; 12 −94 −2; 6 −76 −26; −6 −94 1; −42 −76 −23; −27 −67 −5; 3 −31 7; 3 −10 4; −39 −34 −14; −18 −10 22; −18 −4 −11; 51 −64 1; 30 −67 −11; −27 −67 −8; −42 −31 −11; Decrease: −39 44 −5; −21 50 4	21	2	16/18	None	None	Emotional pictures
30	Blair, K. S., Geraci, M., Otero, M., Majestic, C., Odenheimer, S., Jacobs, M., . . . Pine, D. S	2011	Increase: L dorsal MPFC; R lateral middle frontal cortex; L lateral middle frontal cortex; R medial frontal gyrus; R amygdala; R cingulate gyrus; R postcentral gyrus; R middle temporal gyrus; L middle occipital gyrus; L inferior parietal lobule	Increase: -9 54 24; 29 46 25; -41 37 31; 9 -5 54; 29 -7 -15; 18 5 42; 34 -28 46; 46 -38 -2; -32 -66 17; -43 -24 29	10	0	15/15	8:7/9:6	30.3 (8.49)/31.1 (6.37)	Discourse presentation

31	Koric, L., Volle, E., Seassau, M., Bernard, F. A., Mancini, J., Dubois, B., . . . Levy, R	2012	Increase: Inferior frontal gyrus; Superior frontal gyrus	Increase: 36 21 -18; 12 21 57	2	0	15/15	7:8/6:9	34.3 (3)/34.7 (3)	Cognitive task
32	Gimenez, M., Pujol, J., Ortiz, H., Soriano-Mas, C., Lopez-Sola, M., Farre, M., . . . Martin-Santos, R	2012	Increase: Bil. thalamus and R globus pallidus; R cerebellum and primary visual area; R inferior parietal region;	Increase: 12 − 4 − 5; 42 − 63 − 20	2	0	20/20	5:15/6:14	24.15 (5.22)/24.40 (5.59)	Security review awareness task
33	Gaebler, M., Daniels, J. K., Lamke, J. P., Fydrich, T., & Walter, H	2014	Increase: Supramarginal; Superior temporal; Middle cingulum; Rolandi coperculum; Superior temporal	Increase: –54 –39 27; –63 –48 24; –63 –30 18; –15 –18 42; –12 –30 39; 57 –30 21; 39 –33 21; 57 –30 3	8	0	21/23	5:16/5:18	30.5(7.17)/30.0(7.99)	Emotion regulation task
34	Boehme, S., Ritter, V., Tefikow, S., Stangier, U., Strauss, B., Miltner, W. H., & Straube, T	2015	Increase: left amygdala; right and left insula; mPFC; dorsal part of the ACC; left opercular part of the IFG	Increase: -24 -7 -16; 43 8 -5; -26 15 -7; -6 53 25; -5 8 28; -47 12 11	6	0	16/16	10:6/11:5	29.06(9.84)/30.81(8.83)	Emotion Stroop task
35	Yoon, H. J., Kim, J. S., Shin, Y. B., Choi, S. H., Lee, S. K., & Kim, J. J	2016	Increase: Left inferior frontal gyrus; left DMPFC; left superior temporal gyrus; left TPJ; left PCC; left insula; left dorsomedial thalamus; Increase left DLPFC; right superior frontal gyrus; right inferior frontal gyrus; right ACC; left superior temporal sulcus; right insula; left insula; right cerebellum	Increase: -40 36 -8; -2 62 12; -64 -24 16; -42 -64 18; -2 -64 16; -44 -2 20; -16 -30 12; -38 36 2; 12 -4 74; 40 48 -8; 2 24 34; -58 -32 10; 40 24 8; -44 -4 18; 12 -82 -26	15	0	20/20	10:10/10:10	23.6(2)/23.6(2.3)	Memory task

36	A Richey John, Ghane Merage, Valdespino Andrew, et al	2017	Right posterior cingulate Postcentral gyrus; Right orbital gyrus; Right superior temporal gyrus ; Right subcallosal gyrus; Right medial frontal gyrus ; Right lentiform nucleus ; Left middle occipital gyrus ; Right insula; Left pyramid of vermis; Left precuneus; Left precentral gyrus; Left inferior temporal gyrus ; Left superior temporal gyrus ; Right medial frontal gyrus ; Right postcentral gyrus; Left declive; Right lingual gyrus;<br>Left superior temporal gyrus ; Left middle temporal gyrus; Left insula; Leftsubcallosal gyrus; Right inferior semi-lunar lobule ; Right superior frontal gyrus; Decrease: Right cingulate gyrus; Right medial frontal gyrus Right orbital gyrus; Right cerebellar tonsil; Left superior frontal gyrus ; Right superior parietal lobule ; Left superior frontal gyrus ; Right postcentral gyrus; Right cerebellar tonsil; Increase: Right middle occipital gyrus; Left precentral gyrus; Right parahippocampal gyrus ; Right inferior temporal gyrus ; Left parahippocampal gyrus	Decrease: 23.7 -60.0 13.7; -25.8 -52.2 66.4; 1.5 52.1 -23.8; 54 0.5 -0.6; 21.7 25.0 -17.0; 11.6 -15.5 77.0; 7.6 -6.3 -10.5; -24.7 -96.6 2.0; 39.1 4.1 -18.2; -1.5 -80.3 -41.0; -14.6 -64.3 38.5; -62.1 -13.8 41.2; -43.9 -10.9 -21.5; -53.0 3.6 -0.4; 10.6 70.3 8.6; 31.8 -47.2 69.9; -54 -63.3 -28.1; 22.7 -80.5 -11.2; -54 -10.3 9.8; -55.1 -68.0 8.9; -36.9 -3.5 -2.0; -8 13.1 -13.2; 20.7 -84.8 -56.7; 9.6 7.1 56.4; Decrease: 18.2 17.9 43.1; 24.2 43.4 19.7; 16.2 38.5 -23.5; 21.2 -46.6 -44.4; -17.2 44 30.6; 39.4 -58.1 50.3; -8.1 7.7 55.9; 64.6 -20.3 35.9; 45.5 -49 -60; Increase: 34 -70 4; -27 -27 55; 15 -10 -28; 46 -3 -20; -14 -8 -29	33	3	21/22	13:8/12:10	25.67 (7.61)/26.50 (7.98)	Currency delayed
37	Becker, M. P. I., Simon, D., Miltner, W. H. R., & Straube, T	2017	**Decrease:** left VS; MCC; right ventral AIC	Decrease: -6 14 -5; -6 23 37; 39 8 -11	0	3	16/16	5:11/7:9	35.3 (12.7)/38.4 (12.5)	Time estimation task

Table S2 Study quality evaluated by using modified Newcastle-Ottawa Scale (mNOS)

		**1.Selection**				**2.Comparability**		**3.Exposure**			**4.Statistical analysis**		Note
ID	Authors	1.1 adequate case definition	1.2 representa -tiveness	1.3 controls	1.4 definiti-on of controls	2.1 age & gender	2.2 other variables	3.1 same exposure	3.2 drop-out rate	3.3 behavio-ral manipula -tion check	4.1 p value > 0.001	4.2 false positive correction	
1	Stein, M. B., Goldin, P. R., Sareen, J., Zorrilla, L. T., & Brown, G. G	+	?	+	+	?	+	+	-	+	?	-	
2	Amir, N., Klumpp, H., Elias, J., Bedwell, J. S., Yanasak, N., & Miller, L. S	+	?	+	+	+	+	+	-	+	-	+	
3	Phan, K. L., Fitzgerald, D. A., Nathan, P. J., & Tancer, M. E	+	?	+	+	+	+	+	-	+	-	+	
4	Evans, K. C., Wright, C. I., Wedig, M. M., Gold, A. L., Pollack, M. H	+	+	+	+	+	?	+	-	+	+	+	
5	Gentili, C., Ricciardi, E., Gobbini, M. I., Santarelli, M. F., Haxby, J. V., Pietrini, P., & Guazzelli, M	+	?	+	-	-	?	+	-	+	-	+	

6	Klumpp, H., Angstadt, M., Nathan, P. J., & Phan, K. L	+	?	+	+	+	+	+	-	+	-	+
7	Danti, S., Ricciardi, E., Gentili, C., Gobbini, M. I., Pietrini, P., & Guazzelli, M	+	?	+	+	-	?	+	-	+	-	+
8	Klumpp, H., Angstadt, M., & Phan, K. L	+	?	+	-	+	+	+	-	+	-	+
9	Labuschagne, I., Phan, K. L., Wood, A., Angstadt, M., Chua, P., Heinrichs, M., . . . Nathan, P. J	+	-	+	+	-	+	+	-	+	-	+
10	Frick, A., Howner, K., Fischer, H., Kristiansson, M., & Furmark, T	+	-	+	+	?	+	+	+	+	-	+
11	Ziv, M., Goldin, P. R., Jazaieri, H., Hahn, K. S., & Gross, J. J	+	+	+	+	+	+	+	+	+	-	+
12	Wheaton, M. G., Fitzgerald, D. A., Phan, K. L., & Klumpp, H	+	-	+	+	+	+	+	-	+	-	+

13	Binelli, C., Muniz, A., Subira, S., Navines, R., Blanco-Hinojo, L., Perez-Garcia, D., . . . Martin-Santos, R.	+	+	+	+	+	?	+	+	+	+	+
14	Michalowski, J. M., Matuszewski, J., Drozdziel, D., Koziejowski, W., Rynkiewicz, A., Jednorog, K., & Marchewka, A	+	+	+	+	?	+	+	-	+	-	-
15	Tadayonnejad, R., Klumpp, H., Ajilore, O., Leow, A., & Phan, K. L	+	+	+	+	-	+	+	+	+	+	-
16	Goldin, P. R., Manber-Ball, T., Werner, K., Heimberg, R., & Gross, J. J	+	+	+	+	+	+	+	-	+	-	-
17	Goldin, P. R., Manber, T., Hakimi, S., Canli, T., & Gross, J. J	+	+	+	+	+	+	+	-	+	-	-
18	Shah, S. G., Klumpp, H., Angstadt, M., Nathan, P. J., & Phan, K. L	+	+	+	+	+	-	+	+	+	-	-

19	Nakao, T., Sanematsu, H., Yoshiura, T., Togao, O., Murayama, K., Tomita, M., . . . Kanba, S	+	+	+	+	-	-	+	-	+	-	-
20	Carles Soriano-Mas(Jesus Pujol)	+	?	+	+	+	+	+	+	+	+	+
21	Boehme, S., Mohr, A., Becker, M. P., Miltner, W. H., & Straube, T	+	?	+	+	+	+	+	+	+	+	+
22	Heitmann, C. Y., Feldker, K., Neumeister, P., Zepp, B. M., Peterburs, J., Zwitserlood, P., & Straube, T	+	+	+	+	?	+	+	+	+	+	+
23	Heitmann, C. Y., Feldker, K., Neumeister, P., Brinkmann, L., Schrammen, E., Zwitserlood, P., & Straube, T	+	+	+	+	+	+	+	+	+	+	+

24	Lorberbaum, J. P., Kose, S., Johnson, M. R., Arana, G. W., Sullivan, L. K., Hamner, M. B., . . . George, M. S	+	?	+	+	?	?	+	+	+	-	-
25	Boehme, S., Ritter, V., Tefikow, S., Stangier, U	+	?	+	+	?	+	+	+	+	-	+
26	Bunford, N., Kujawa, A., Fitzgerald, K. D., Monk, C. S., & Phan, K. L	+	+	+	+	-	+	+	+	+	+	+
27	Quadflieg, S., Mohr, A., Mentzel, H. J., Miltner, W. H., & Straube, T	+	+	+	+	+	-	+	-	+	+	+
28	Blair, K. S., Geraci, M., Hollon, N., Otero, M., DeVido, J., Majestic, C., . . . Pine, D. S	+	+	+	+	+	+	+	-	+	?	?
29	Bruhl, A. B., Rufer, M., Delsignore, A., Kaffenberger, T., Jancke, L., & Herwig, U	+	+	+	+	+	?	+	-	+	+	+

30	Blair, K. S., Geraci, M., Otero, M., Majestic, C., Odenheimer, S., Jacobs, M., . . . Pine, D. S	+	+	+	+	+	?	+	-	+	+	+
31	Koric, L., Volle, E., Seassau, M., Bernard, F. A., Mancini, J., Dubois, B., . . . Levy, R	+	?	+	+	+	+	+	-	+	?	?
32	Gimenez, M., Pujol, J., Ortiz, H., Soriano-Mas, C., Lopez-Sola, M., Farre, M., . . . Martin-Santos, R	+	+	+	-	+	+	+	-	+	?	?
33	Gaebler, M., Daniels, J. K., Lamke, J. P., Fydrich, T., & Walter, H	+	+	+	+	+	+	+	+	+	?	?
34	Boehme, S., Ritter, V., Tefikow, S., Stangier, U., Strauss, B., Miltner, W. H., & Straube, T	+	+	+	+	+	-	+	+	+	+	+
35	Yoon, H. J., Kim, J. S., Shin, Y. B., Choi, S. H., Lee, S. K., & Kim, J. J	+	-	+	?	+	+	+	-	+	+	+

36	A Richey John, Ghane Merage, Valdespino Andrew, et al	+	?	+	?	+	+	+	-	-	+	+
37	Becker, M. P. I., Simon, D., Miltner, W. H. R., & Straube, T	+	+	+	?	+	+	+	-	+	+	+
	**K agreement**	**1**	**0.89**	**1**	**0.86**	**0.89**	**0.86**	**1**	**0.78**	**1**	**0.95**	**0.73**
	**mean K agreement**	**0.953**										
